# Gut Microbiota Analysis in Silkworms (*Bombyx mori*) Provides Insights into Identifying Key Bacterials for Inclusion in Artificial Diet Formulations

**DOI:** 10.3390/ani14091261

**Published:** 2024-04-23

**Authors:** Lei Xin, Yazhen Chen, Wantao Rong, Yingcan Qin, Xiaodong Li, Delong Guan

**Affiliations:** 1 Guangxi Key Laboratory of Sericulture Ecology and Applied Intelligent Technology, Hechi University, Hechi 546300, China; xinlei@hcnu.edu.cn (L.X.); cyz2013060415@163.com (Y.C.); 18023@hcnu.edu.cn (W.R.); tanyingcan1@hcnu.edu.cn (Y.Q.); 2 Guangxi Collaborative Innovation Center of Modern Sericulture and Silk, Hechi University, Hechi 546300, China

**Keywords:** silkworm, 16S rRNA sequencing, gut microbiome, artificial diets

## Abstract

**Simple Summary:**

Silkworms are monophagous insects that primarily feed on mulberry leaves. However, in sericulture, cost-effective artificial diets are often used to maximize productivity, which can lead to dietary stress, delayed development, and reduced silk quality in silkworms. Improving silkworms’ adaptability to artificial diets is crucial for the sericulture industry. This study aimed to identify key intestinal bacteria taxa that facilitate silkworms’ adaptation to dietary transitions by comparing the gut microbiomes of silkworms reared on three different diets: exclusive mulberry leaves, exclusive artificial diet, and a sequential transition from artificial diet to mulberry leaves. Using advanced sequencing techniques and bioinformatic analyses, we discovered that the transitional diet group harbored an intermediary gut microbiota complexity, with specific bacterial groups, such as *Lactobacillus* and *Weissella*, potentially aiding in the adaptation process. Our findings provide valuable insights into the adaptation mechanisms of silkworm gut microbiota in response to dietary changes. These results pave the way for developing tailored probiotic supplements that can be added to artificial diets, enhancing silkworms’ adaptability and overall health without altering the diet composition. This research holds promise for optimizing sericulture practices, improving silk quality, and ensuring the sustainability of the silk industry.

**Abstract:**

The gut microbiome significantly influences the health and productivity of silkworms (*Bombyx mori*), the cornerstone of sericulture. With the increasing use of cost-effective artificial diets in sericulture, it is crucial to understand how these diets impact the silkworm gut microbiomes. Here we employed 16S rRNA sequencing to delineate the impact of three distinct dietary regimens on the silkworm gut microbiomes: exclusive mulberry leaf diet (SY), exclusive artificial feed diet (SL), and a sequential transition from artificial feed to mulberry leaves (ZS). Our results unveiled stark differences in microbial diversity across the groups, with the ZS group displaying an intermediary complexity. LefSe and random forest analyses identified Methylobacteriaceae, *Microbacterium*, and *Rhodococcus* as significantly enriched in the ZS group, suggesting their potential to facilitate silkworms’ adaptation to dietary transitions. Functional profiling revealed differential pathway regulation, indicating a metabolic reconfiguration in response to dietary modulations. Notably, the enrichment of *Lactobacillus* and *Weissella* in both the SL and ZS groups highlights their potential as probiotics in artificial diets. Our findings provide insights into the diet adaptation mechanisms of silkworm gut microbiota, paving the way for harnessing the intestinal bacteria to enhance silkworm health and silk production through targeted microbial interventions in sericulture practices.

## 1. Introduction

Sericulture, the craft of cultivating silkworms (*Bombyx mori*) to produce silk, has been a fundamental component of the global textile industry for thousands of years. This domesticated insect has been subjected to intensive artificial selection, leading to the development of a highly specialized silk-producing entity [1,2]. Nonetheless, the commercial success and sustainability of sericulture face ongoing challenges, necessitating the optimization of rearing environments alongside the minimization of production expenses [3,4]. A significant factor in silkworm husbandry that has recently attracted considerable interest is the intricate relationship between diet, gut microbiota, and the health of the host organism [5,6,7].

As monophagous insects, silkworms (*Bombyx mori*) predominantly consume mulberry leaves (*Morus alba*) in their natural habitat, which are crucial for their nutritional intake [4,8]. The nutrient composition of mulberry leaves, particularly the balance of proteins, carbohydrates, and essential micronutrients, plays a pivotal role in the growth, development, and silk production of silkworms [4,8,9]. However, the increasing demand for silk and the need for cost-effective and efficient production methods have led to the widespread adoption of artificial diets in commercial sericulture [6,10,11,12]. These formulated feeds, often based on a combination of soybean meal, corn powder, and various nutritional supplements, aim to provide a balanced and easily digestible nutrient profile for optimal silkworm growth [11,12]. Although artificial diets have bolstered productivity and lessened the industry’s dependence on mulberry farming, they have also engendered novel challenges concerning the health of silkworms and the caliber of silk produced [13,14,15].

The gut microbiota of silkworms, which encompasses a myriad of bacterial species, is of paramount importance for the digestion of nutrients, regulation of the immune system, and the general well-being of the host [6,13,16]. The dietary habits of the host substantially sway the composition and functionality of the gut microbiota, given that varying dietary inputs furnish unique substrates that promote the growth and metabolic activities of different microbes [6,16,17]. Studies have demonstrated that the transition in silkworms from a diet dominated by mulberry leaves to one consisting of artificial formulations can lead to marked changes in the gut microbiota’s architecture [6,18]. These dietary-driven alterations, involving phyla such as Proteobacteria and Firmicutes, particularly *Lactobacillus*, can significantly impact silkworm physiology. Specific microbial groups may play a role in the digestion of intricate dietary substances, the generation of indispensable nutrients, and the regulation of immune reactions [6,16,18].

Although the significance of gut microbiota for the health and productivity of silkworms is increasingly acknowledged, the nuanced mechanisms through which disparate dietary regimens sculpt the structure and functionality of the microbial community are still largely a mystery. Extant research has predominantly concentrated on contrasting the gut microbiomes of silkworms that are either reared on mulberry leaves or are fed artificial diets [6,14,16,19,20]. These investigations have shed light on the general differences in microbial makeup between these two dietary cohorts, highlighting the dominance of bacteria such as Proteobacteria, Firmicutes, Bacteroidetes, and Actinobacteria in the silkworm intestines. However, they have not thoroughly examined the potential restorative responses that occur when silkworms revert to a natural mulberry leaf diet [6,16]. Additionally, pinpointing the specific microbial taxa that could positively influence silkworm health and silk production across varying dietary conditions continues to be an unresolved challenge.

To bridge this knowledge gap, we propose a novel experimental approach that incorporates a third dietary group: a sequential transition from an artificial diet to a mulberry leaf-based diet (ZS). By comparing the gut microbiota of ZS with the silkworms reared exclusively on mulberry leaves (SY), and those fed solely on an artificial diet (SL), we aim to elucidate the adaptive capacity of the gut microbiomes in response to dietary perturbations. Despite the prevalence of artificial diets in commercial silkworm rearing, the inclusion of the ZS group is strategic, as it may offer insights into enhancing the formulation of artificial diets. Given that silkworms are naturally adapted to mulberry leaves, identifying microbial taxa that consistently correlate with superior silkworm outcomes across various diets, especially within the ZS group, could be instrumental. This could form the basis for developing targeted microbial interventions to improve the efficacy and quality of artificial diets and aid in the recovery of silkworms from the effects of artificial diets, a factor critical for the sustainability of sericulture. We posit that the ZS group will present a microbial community structure that is intermediate between the SY and SL groups, indicative of the gut microbiota’s inherent resilience and adaptability in response to fluctuating dietary conditions.

To encapsulate, the objective of this study is to furnish a thorough comprehension of the dynamic interplay among diet, gut microbiota, and the health of the silkworm host, with a particular emphasis on the gut microbial community’s adaptive responses to dietary transitions. Through the comparative analysis of the gut microbiomes across silkworms raised on exclusive mulberry leaves, artificial diets, and a transitional diet, this research has yielded concrete evidence of the adaptability and resilience of the silkworm gut microbiota when subjected to dietary changes. The recognition of pivotal microbial taxa linked to each dietary regimen has set the stage for subsequent inquiries into the functional roles these microbes play in the physiology and performance of silkworms. Furthermore, the study underscores the promising potential of targeted microbial interventions—encompassing probiotics and microbial inoculants—as a strategy to augment the health and productivity of silkworms, without necessitating alterations to the foundational composition of the artificial diet. This innovative approach may well be the key to refining sericulture practices, elevating the quality of silk, and safeguarding the long-term sustainability of the silk industry.

## 2. Materials and Methods

### 2.1. Samples and Diets

The silkworm samples used in this study were collected from the Gui Can No. 5 strain, which has been under long-term cultivation in the laboratory at Hechi University. Each of the three experimental groups comprised 50 specimens, all initiated under identical physiological conditions. Before the feeding stage, the larvae were kept in a sterile environment, devoid of any prior exposure to food sources. Throughout the rearing period, the silkworms were nurtured up to the third day reaching the fifth instar stage. Specifically, the group that transitioned from artificial feed to mulberry leaves underwent this dietary change at the third instar, with the transition meticulously timed to the second day of reaching this stage.

Following a 24-h starvation period, ten silkworms from each rearing method were randomly selected for dissection. The midgut was specifically targeted for analysis due to its central role in harboring the core gut microbial community and its common use in previous studies [16,21,22]. The dissection process was performed on a sterile bench to maintain aseptic conditions. Before dissection, the larval bodies were surface sterilized with 75% ethanol to minimize the risk of external microbial contamination. The sterilization procedure allowed for the precise isolation and removal of the complete midguts without introducing extraneous microbial populations. The harvested midguts were placed on ice and the Malpighian tubules and fat bodies were quickly removed. The cleaned midgut samples were then transferred to pre-chilled 2 mL sterile centrifuge tubes and stored at −80 °C until DNA extraction. For each experimental group, this procedure was performed on five independent biological replicates, each using midguts pooled from 10 larvae.

The low-cost artificial diet used in this study was formulated to meet the nutritional requirements of silkworms while minimizing production costs. The diet composition included defatted soybean powder (36.782%), corn flour (30%), defatted rice bran (11.423%), rapeseed meal (8%), inorganic salt mixture (2.669%), vitamin mixture (0.235%), soybean oil (1.779%), phytosterol (0.204%), ascorbic acid (1%), citric acid (3%), carrageenan (4%), and preservatives (0.91%). This formulation aimed to provide a balanced mix of proteins, carbohydrates, lipids, vitamins, and minerals to support silkworm growth and development.

### 2.2. DNA Extraction and 16S rRNA Gene Sequencing

Total DNA was isolated from the midgut samples using a hexadecyl trimethyl ammonium bromide (CTAB)/sodium dodecyl sulfate (SDS) based protocol. DNA samples were quantified using the NanoDrop™ spectrophotometer (Thermo Fisher Scientific, Wilmington, DE, USA). The V3–V4 hypervariable region of the 16S rRNA gene was then PCR amplified using universal primers. The forward primer sequence used was ACTCCTACGGGAGGCAGCA, and the reverse primer sequence was GGACTACHVGGGTWTCTAAT. The selection of these primers was informed by a wealth of research validating their efficacy and dependability across diverse tissue types [23,24,25]. The PCR products were subjected to verification through 2% agarose gel electrophoresis, followed by SYBR Green staining, before undergoing purification, quantification, and pooling. Sequencing was conducted on an Illumina MiSeq platform with paired-end technology (2 × 300 bp), facilitated by the Shanghai Yaoen Biotechnology company (Shanghai, China).

To prepare DNA libraries suitable for Next-Generation Sequencing (NGS), the TruSeq Nano DNA LT Library Prep Kit (Illumina, Inc., San Diego, CA, USA) was utilized. Amplicons from the V3–V4 region of the 16S rRNA gene were used to prepare the metagenomic libraries. The amplicon DNA was initially subjected to fragmentation, end-repair, and the addition of a single ‘A’ nucleotide (A-tailing) following the manufacturer’s guidelines. This process facilitates the subsequent ligation of adapters, which are designed with a single ‘T’ nucleotide overhang. Post-ligation, the library fragments were selectively amplified through a limited number of PCR cycles. This enrichment step was executed using the Agilent Bioanalyzer system (Agilent Technologies, Santa Clara, CA, USA). The anticipated size range for the amplicons, as delineated by the primer binding sites, spanned from 292 to 480 base pairs (bp).

### 2.3. Bioinformatic and Statistical Analysis

We initially processed paired-end raw reads from 30 samples using Trimmomatic v0.38 [26] to remove low-quality sequences. This step was crucial for the removal of low-quality sequences, including those containing adapter sequences, contaminants, or with a Phred quality score below 20—indicative of base-calling accuracy falling short of 90%. Furthermore, any reads that were shorter than 50 base pairs were also eliminated. The high-quality reads that remained were then advanced to a thorough bioinformatics analysis facilitated by QIIME II (version 2.01) [27]. Within the QIIME II framework, we harnessed the DADA2 pipeline [28] to execute a series of processes, including primer trimming, sequence filtering, error correction (denoising), merging of paired-end reads, and the elimination of chimeric sequences across each independent library. The “cut-adapt trim-paired” utility within QIIME was instrumental in the removal of primer sequences and the exclusion of any sequences that did not match the required criteria. The “DADA2 denoise-paired” command further refined the quality control measures.

Post the denoising process, amplicon sequence variants (ASVs) were compiled into a comprehensive feature table, with singleton ASVs—those occurring only once across all samples—being systematically excluded. An R script was employed to meticulously evaluate the distribution of sequence lengths among the remaining high-quality sequences. In alignment with the current QIIME2 standards, these ASVs were subsequently allocated into the designated study cohorts: SY, which was exclusively reared on mulberry leaves; SL, which was fed an artificial diet; and ZS, which underwent a dietary transition from artificial feed to mulberry leaves during the third to fifth instar stages. Utilizing QIIME2, a suite of indices was applied to assess alpha diversity within the microbial communities. These included the Chao1 and Observed Species indices to gauge community richness, the Shannon and Simpson indices for quantifying diversity, Faith’s Phylogenetic Diversity as a measure of evolutionary divergence, Pielou’s evenness for assessing community uniformity, and Good’s coverage to ascertain the thoroughness of our sampling efforts.

To discern the structural intricacies within the microbial communities, we deployed the linear discriminant effect size (LefSe) analysis coupled with random forest estimation, both facilitated by the PersonalBio online platform (https://www.genescloud.cn/ accessed on 17 January 2024). The Principal Coordinate Analysis (PCoA) was executed utilizing a rarefied ASV table, which was instrumental in generating the distance matrices through the “qiime diversity core-metrics-phylogenetic” command. The functional profiling of the hypothesized Kyoto Encyclopedia of Genes and Genomes (KEGG) metabolic pathways was approached using the PICRUSt2 software (https://github.com/picrust/picrust2 accessed on 22 April 2024) [29]. To substantiate the robustness of our analytical findings, statistical significance, encompassing *p*-values and false discovery rates (FDRs), was rigorously determined with the aid of the *p* analysis and *p* adjust tools available on the PersonalBio online platform (https://www.genescloud.cn/ accessed on 15 February 2024).

## 3. Results

### 3.1. Alpha and Beta–Diversity

We quantitatively assessed the bacterial biodiversity of silkworms subjected to different feeding regimens using various diversity indices. The feeding regimens included an exclusive mulberry leaf diet (SY), an exclusively formulated feed diet (SL), and a sequential diet transitioning from formulated feed to mulberry leaves (ZS). The primary metrics used for biodiversity evaluation were the Chao1, Simpson, Shan-non, Pielou’s evenness (Pielou_e), observed species, Faith’s phylogenetic diversity (Faith_pd), and Goods coverage indices. Rarefaction curves displaying the alpha diversity indices for each sample are presented in Supplementary Figure S1. These curves plateau beyond a sequencing depth of 20,000, nearly leveling off, which signifies that the sequencing depth was adequate to capture the diversity present within the samples. This leveling indicates that additional sequencing would not likely yield a significant number of undiscovered amplicon sequence variants (ASVs). The stabilization of these curves provides strong evidence of both the sufficiency of the sample sizes and the credibility of the data.

**Figure 1:**
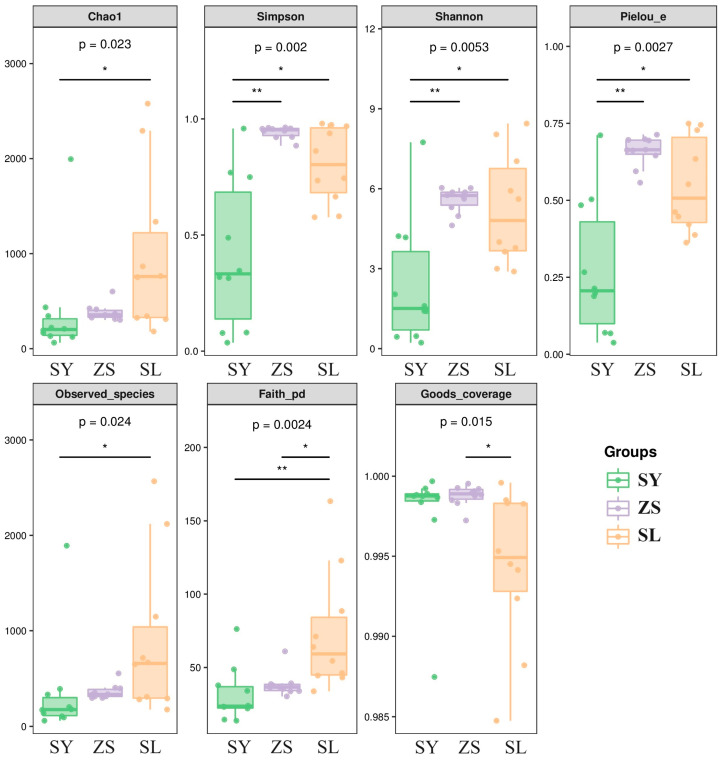
Grouped Boxplot of Alpha Diversity Indices. Each panel corresponds to a specific alpha diversity index, indicated by the shaded grey area at the top. Within each panel, the *x*-axis represents the grouping labels, while the *y*-axis represents the corresponding values of the alpha diversity index. In the boxplot, the symbols are interpreted as follows: the upper and lower lines of the box represent the interquartile range (IQR); the line within the box represents the median; the upper and lower whiskers represent the minimum and maximum values falling within 1.5 times the IQR; points outside the whiskers represent outliers. The numbers beneath the labels of diversity indices denote the *p*-values from the Kruskal-Wallis test. An asterisk (*) signifies statistical significance at the *p* < 0.05 threshold, while two asterisks (**) denote high statistical significance at the *p* < 0.01 threshold.

In general, the SY regimen recorded a lower Chao1 index compared to the SL group, as illustrated in Figure 1 and Supplemental File S1. The Simpson index, which measures both the dominance and evenness of species within a community, nearly reached unity across the majority of samples, pointing to a rich diversity within these populations. The ZS silkworms, which underwent a dietary transition, consistently showed high Simpson diversity indices, indicative of a complex and well-balanced microbial ecosystem. When assessing the intricacy of community structure across different dietary treatments, Shannon’s index—considering both species abundance and evenness—highlighted considerable variations. Notably, the SL group’s silkworms presented the highest diversity levels, a finding in marked contrast to the SY group with the lowest diversity. This comparison accentuates the pronounced impact of diet on the community’s complexity. Further scrutiny revealed that the observed species count in the SL group aligned with the Chao1 predictions, inferring a more biodiverse community within this group, as depicted in Figure 1 and elaborated in Supplemental File S1.

Conversely, the SY group exhibited a lower tally of observed species. In terms of Faith’s phylogenetic diversity index, a wide spectrum was observed across groups, with the SL group generally manifesting higher Faith_pd values, suggesting a more phylogenetically diverse microbial assemblage when compared to the SY group. This not only speaks to the variety of species but also to the diversity of their evolutionary backgrounds. Additionally, the Good’s coverage index was remarkably high for all samples, signifying that our sampling efforts were exhaustive and that the species present within the communities were well-represented. With Good’s coverage indices consistently surpassing 0.984 and many samples reaching above 0.998, the high degree of coverage confirms the strength of our sampling strategy, as further detailed in Figure 1 and Supplemental File S1.

**Figure 2:**
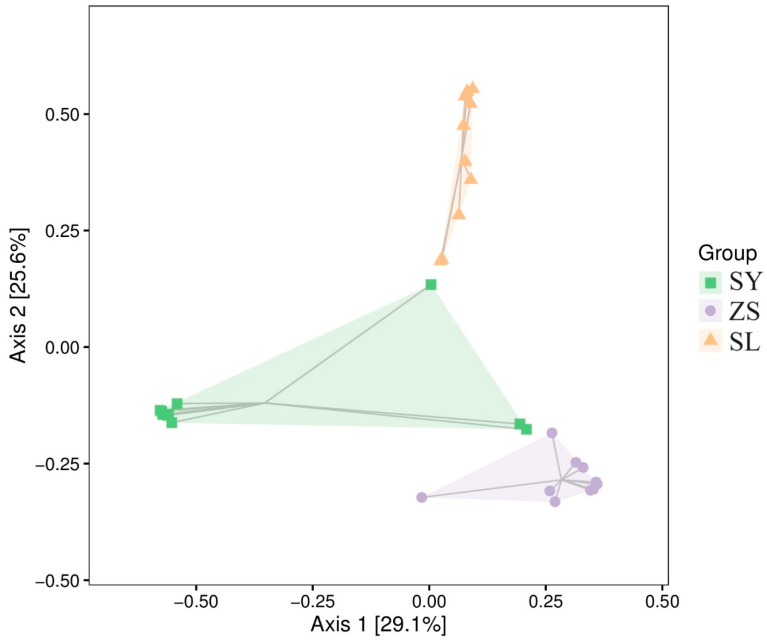
Two-Dimensional Ordination Plot of PCoA Analysis on Bray-Curtis distance matrix. Each point in the plot represents a sample, with distinct colors indicating different samples (groups). The percentages within the parentheses of the axes represent the proportion of sample dissimilarity data (distance matrix), explained by the corresponding axis.

In our quest to investigate beta diversity across the experimental groups, we engaged an analysis based on Bray-Curtis dissimilarity metrics, which effectively highlighted the compositional divergence among the distinct dietary regimes (as detailed in Supplementary File S2). The application of Principal Coordinates Analysis (PCoA) using the Bray-Curtis distance matrix offered a particularly insightful depiction of our three-tiered dataset, achieving a clear differentiation between the groups. It is worth noting that the initial two axes of the PCoA accounted for a substantial proportion of the dataset’s variability, with 29.1% and 25.5% respectively (Figure 2).

Utilizing the distance matrix for comparative analysis among groups, we discerned significant variations in the internal dissimilarity indices of silkworm cohorts under different dietary conditions. The cohort that was exclusively fed a mulberry leaf diet (SY) predominantly showed internal dissimilarity indices below the 0.3 mark, suggesting a relatively uniform microbial community within this group. Conversely, the group that was solely administered formulated feed (SL) presented a wider array of within-group dissimilarity scores, indicative of greater heterogeneity. This variability within the SL group implies a varied response to the artificial diet, as further elaborated in Supplementary File S2. The ZS group, which experienced a transition between diets, exhibited intermediate dissimilarity levels, suggesting that dietary shifts could potentially mitigate community dissimilarity. Additionally, cross-group analyses revealed elevated dissimilarity indices, underscoring the substantial effect of diet on microbial community composition. A pronounced divergence was particularly observed between the SY and SL groups, with dissimilarity indices approaching unity, signifying clear distinctions attributed to the uniqueness of their respective diets. This finding emphasizes the significant role of dietary composition in shaping the gut microbiota structure within these silkworm populations.

Similar patterns emerged when comparing the ZS group to both the SY and SL groups, with the dissimilarity metrics revealing a clear segregation based on dietary practices. Although the sequential diet (ZS) ostensibly acted as an intermediary between the dietary extremes, it undeniably engendered a distinctive compositional profile, as evidenced by the pairwise distance measurements. These collective observations underscore the profound influence of diet on the microbial community’s compositional signatures within the sericulture populations studied.

### 3.2. Structural Diversity of Silkworms’ Midgut Microbiota

**Figure 3:**
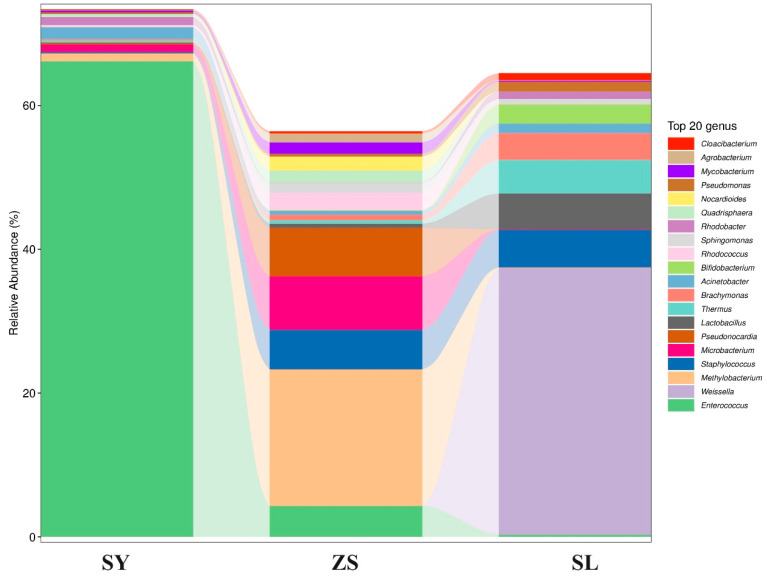
Bar Chart of Species Composition at Genus Level. The chart illustrates the top 20 abundant species within each genus.

To gain a comprehensive understanding of the significant influence of different diets on the gut microbiomes of silkworms, we conducted a comparative analysis of abundant microbial species within the feeding groups (Figure 3, Supplementary File S3). Comparative analysis revealed that the SY group harbored less diverse but more specialized bacterial communities, potentially due to the specific nutrients and fibrous content of mulberry leaves. In contrast, the SL group supported a more heterogeneous bacterial population, indicative of the varied ingredients present in the artificial feed that may accommodate a broader spectrum of bacterial taxa. Notably, the ZS feeding regimen exhibited an intermediary microbial community complexity, implicating the interplay between initial exposure to a formulated diet and the gut microbiota’s adaptability to subsequent natural dietary input.

The bacterial community composition within the gut of the SY silkworm group was predominated by organisms from the phylum Firmicutes, particularly within the class *Bacilli*. Notably, the predominant genera included *Enterococcus*, which is commonly found in various gastrointestinal tracts, and *Staphylococcus*, an opportunistic pathogen frequently encountered in the environment. Additionally, *Lactobacillus*, widely recognized for its roles in fermentation processes and as a significant gut commensal, was also a prominent member of this community, as depicted in Figure 3.

In contrast, the ZS feeding regimen presented a more diverse bacterial profile. Notably, *Methylobacterium* from the phylum Proteobacteria was predominant, followed by an increased prevalence of *Microbacterium* from the phylum Actinomycetes. Meanwhile, the SL regime was characterized by a distinctly different microbial community structure, rich in the phylum Proteobacteria. Within this group, *Weissella* and various unnamed *Enterobacteriaceae* were notably prominent. This microbial composition contrasts starkly with that of the SY group, underscoring the significant influence of dietary composition on the configuration of the gut microbiota, as illustrated in Figure 3.

Building upon our analysis, we utilized the exhaustive abundance data garnered from all samples to establish an inference network. This network was crafted to delineate the interspecies relationships among the constituents of the microbial community. We then scrutinized this network to ascertain the presence of any discrete modular units within the microbiome. Furthermore, we aimed to identify any keystone species—those whose variations could significantly influence the overall microbial composition.

**Figure 4:**
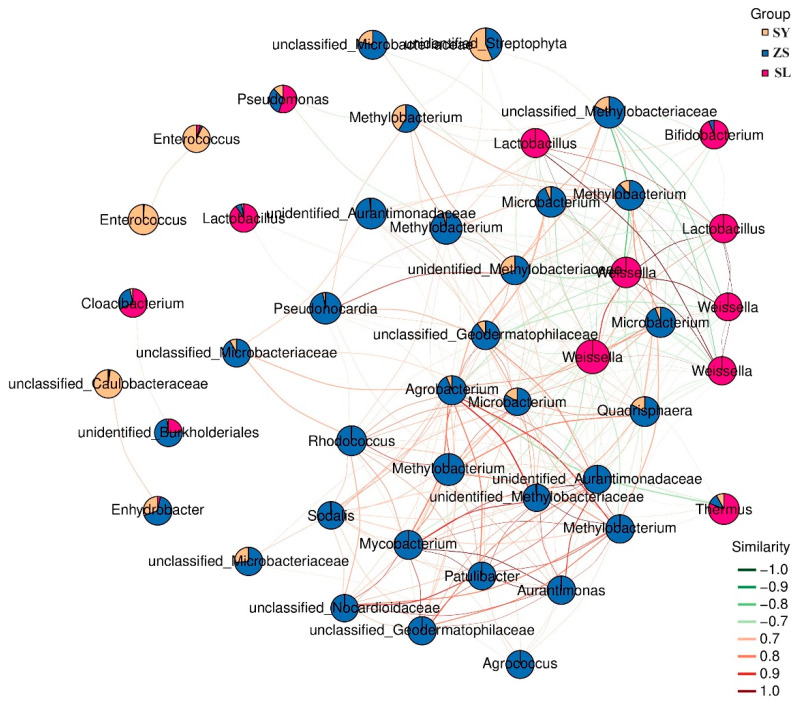
Dominant Species Sub-Network Graph with Grouped Abundance Pie Charts. Each node represents an ASV within a sample, where the size of the node is proportional to its abundance in log2(CPM/n) units. The graph displays the top 50 ASVs/OTUs based on average abundance across samples. The pie charts depict the relative abundance of each node across different groups. The edges between nodes indicate correlations, with red lines denoting positive correlations and green lines denoting negative correlations.

Utilizing the top 50 nodes by average abundance, which encompassed all taxa surpassing 1% across samples, we developed a subnetwork focused on dominant species. This approach served to simplify the graphical representation while maintaining clarity. The resultant network corroborated our previous compositional analyses, offering a more visually accessible interpretation. Distinct clusters emerged, each characterized by a key dominant genus (Figure 4). Specifically, *Enterococcus* remained predominant in SY, followed by an unclassified Caulobacteraceae. The pivotal role of *Weissella* within SL was repeatedly highlighted, demonstrating broad associations with ZS. ZS nodal composition and distribution remained complex; trends favoring *Methylobacterium* and *Microbacterium* were unchanged. However, precise genera within *Proteobacteria* and *Actinobacteria*, including Agrococcus and Pseudonocardia, also emerged as notable.

Additionally, examining dominant microbial proportions across groups revealed most were unique to each. Despite greater ZS-SL connectivity, dominant microbes within ZS were more closely related to SY, including several high-abundance taxa shared between both groups, such as *Methylobacterium* and unidentified_*Streptophyta*. Searching for genus abundantly present across all three groups discerned noteworthy *Pseudomonas*, *Thermus*, and *Lactobacillus*. These findings may indicate certain functional attributes shared among microbiomes, potentially revealing an underlying resilience mechanism or shared adaptive strategy.

### 3.3. Species Variation Analysis and Biomarker Identification

Capitalizing on our preliminary observations regarding the abundance and interrelationships of gut microbiota across different rearing strategies, we proceeded to perform a sophisticated analysis. This involved the application of Linear Discriminant Analysis Effect Size (LefSe) in conjunction with Random Forest techniques. The objective was to meticulously discern the distinctions between the groups and to identify biomarker taxa with statistical robustness.

The LefSe analysis yielded clear distinctions among the biomarkers present across the various dietary and environmental groups. Within the SL group, there was a pronounced prevalence of Firmicutes, notably from the classes *Bacilli* and *Clostridia*. The order Lactobacillales, encompassing genera such as *Lactobacillus* and *Streptococcus*, showed marked dominance. This was reflected in the LDA scores, which varied between 3.74 and 4.43, and were accompanied by low *p*-values, attesting to their robust statistical significance. Furthermore, the phylum Bacteroidetes, including the genus *Bacteroides*, was also found to be abundant, implying a diet that fosters a diverse array of fermentative and fiber-degrading microbial species.

**Figure 5:**
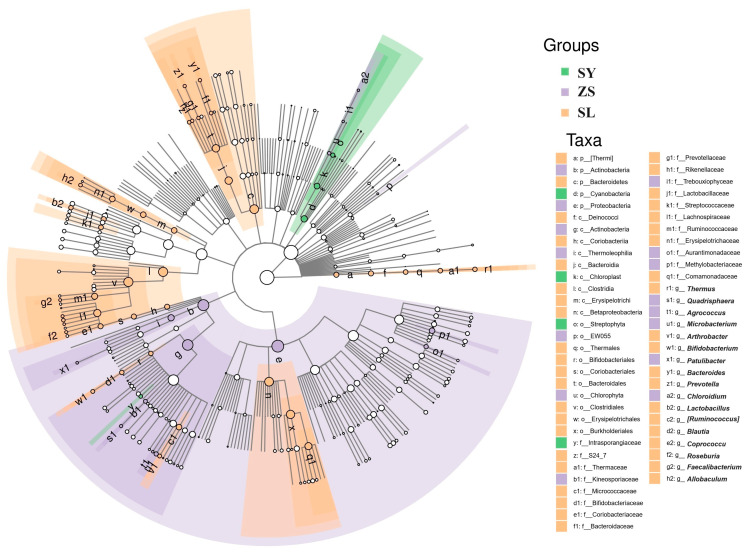
Differential Taxonomic Units Displayed of LefSe analysis on a Taxonomic Hierarchy Tree. The cladogram displays the taxonomic relationships of major taxonomic units from phylum to genus (from inner circle to outer circle) within the community of samples. Node sizes correspond to the average relative abundance of each taxonomic unit. Hollow nodes represent taxonomic units with non-significant inter-group differences, while nodes of various colors indicate taxonomic units that exhibit significant inter-group differences, with higher abundance in the respective colored group of samples. Letters identify the names of taxonomic units showing significant inter-group differences.

The ZS group exhibited a marked abundance of Proteobacteria and Actinobacteria, including Methylobacterium from the Rhizobiales order and *Microbacterium*, with the highest LDA score recorded at 5.15, reflecting a distinct microbiota possibly adapted to a specialized or transitional dietary regimen. These bacteria are often associated with a more dynamic environmental interaction, potentially linked to varied dietary sources. The SY group was characterized by a significantly higher abundance of Cyanobacteria, particularly from the Chloroplast lineage, including taxa such as *Chlorophyta* and *Trebouxiophyceae*, with LDA scores around 4.85. (Figure 5, Supplementary File S4).

**Figure 6:**
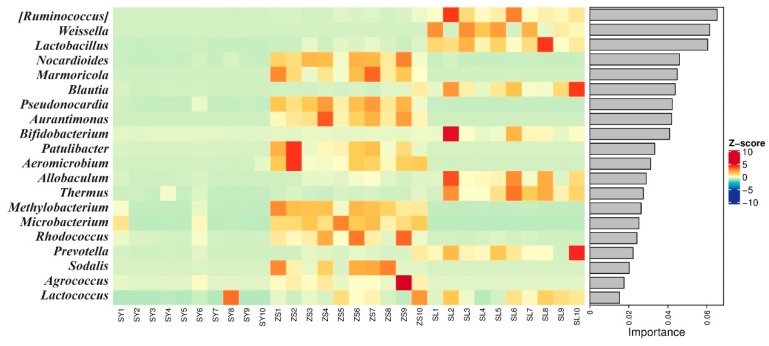
Heatmap of the Top 20 ASV/Genus in terms of Importance. The horizontal axis represents the importance score of species for the classification model, while the vertical axis denotes the ASV/Genus names. Species are arranged from top to bottom based on their decreasing influence on group differentiation. The top-ranking species are considered indicative of inter-group differences.

Random forest analysis elucidated *Bifidobacterium*, *Allobaculum*, and *Thermus* emerged strongly affiliated with the SL regimen, each exhibiting a distinctive abundance pattern versus other diets. *Ruminococcus* was notable for its preeminence in SL, registering the highest feature importance score of 0.065 (Figure 6, Supplementary File S5). Substantial *Ruminococcus* abundance in SL underscores its potentially critical role in high-fiber feed component catabolism. Additionally, the prominence of *Weissella* and *Lactobacillus* in the SL group corresponds with previous insights into their importance and prevalence in sericultural settings. In contrast, the SY group exhibited a microbiota signature that was notably different from both the ZS and SL groups (Figure 6).

The ZS group showcased a striking shift in bacterial composition, with a notable enrichment of *Methylobacterium* and *Rhodococcus* (Figure 6). These changes in microbial abundance highlight the responsive adaptations of the gut microbiota to the altered dietary conditions. Moreover, the ZS group exhibited a marked preference for Actinomycetes, including Nocardioides, Marmoricola, and Pseudonocardia, which are known to thrive in close association with *Methylobacterium* and *Rhodococcus*. The prominence of these Actinomycetes in the ZS group underscores the gut microbiota’s remarkable capacity to adapt to the gradual introduction of mulberry leaves into the diet (Figure 6). Despite the inherent complexity of the ZS gut microbiota, a discernible inclination towards Actinomycetes representation was evident. These findings not only corroborate the flexibility and adaptability of the gut microbiota in response to dietary alterations but also underscore the intricate interplay between dietary composition and the structure of the microbial community.

### 3.4. Functional Differentiation of Gut Microbiota between Feed-Fed and Mulberry Leaf-Fed Silkworms

**Figure 7:**
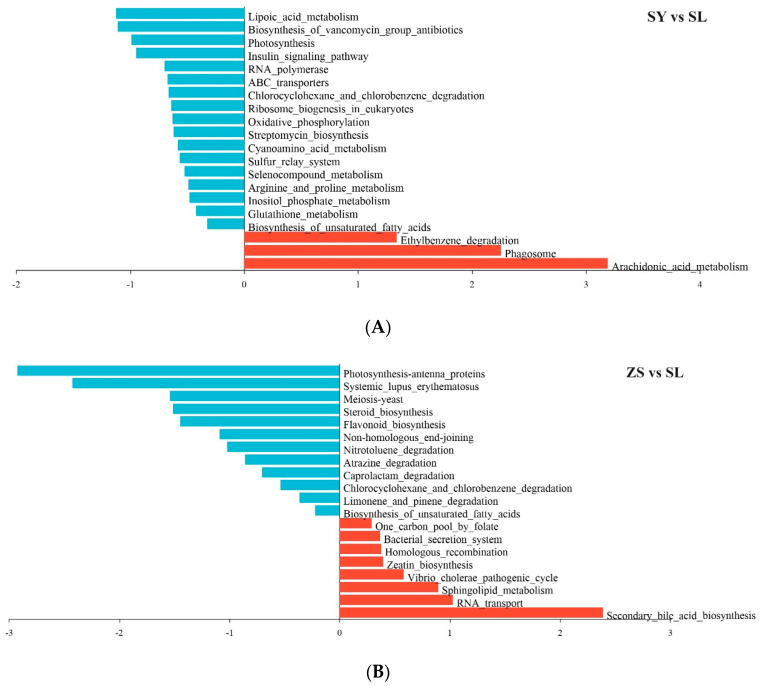
KEGG Enrichment Analysis among Comparison Groups of SY vs. SL (**A**) and ZS vs. SL (**B**). The log fold change (logFC) values denote the relative expression changes between the two groups, with negative values indicating a decrease and positive values indicating an increase in the latter group relative to the former group.

With the identification of biomarker gut microbiota across various silkworm-rearing conditions, we proceeded to perform a comparative functional metagenomic analysis. A significant upregulation in ethylbenzene degradation, with a 1.338 log2 fold change (logFC), was observed in the SL group compared to the SY group. This suggests an enhanced catabolic capacity attributed to the composition of the artificial feed. Concurrently, there was a downregulation in the SL group for photosynthesis and insulin signaling pathways by −0.987 and −0.9453 logFC, respectively. This points towards a reduction in phototrophic metabolism and a modulation in glucose homeostasis. Furthermore, the SL group exhibited suppressed levels of streptomycin biosynthesis (−0.6183 logFC), oxidative phosphorylation (−0.6264 logFC), and fatty acid biosynthesis (−0.3223 logFC), aligning with a metabolic adaptation towards feed-centric nutrition. In contrast, an upregulation of the phagosome pathway by 2.253 logFC in the SL group highlights the integral role of gut microbiota in the host’s immune response (Figure 7A, Supplement File S6).

Comparing SL and ZS, several SL pathways were downregulated, including meiosis (−1.539 logFC) and steroid biosynthesis (−1.51 logFC). Photosynthesis antenna proteins decreased substantially (−2.923 logFC), reflecting dietary evolution away from phototrophic elements. RNA transport increased by 1.028 logFC alongside secondary bile acid biosynthesis (2.388 logFC), assuming adaptive progression tailoring microbial machinery to synthesized diets. Microbial secretion systems marginally increased (0.3666 logFC) with slight increments in recombination and metabolism pathways, implying subtleties in genomic stability and physiology. The upregulation of Vibrio cholerae pathogenic cycle (0.5805 logFC) warrants further health implications of diet modification (Figure 7B, Supplement File S6).

## 4. Discussion

In the realm of sericulture, understanding the complex dynamics of the silkworm gut microbiomes and their response to dietary modulations is crucial for optimizing silk production and ensuring the sustainability of the industry. While silkworms have undergone extensive domestication and are primarily reared in captive environments, their gut microbiota still plays a vital role in mediating host adaptation to dietary changes. Our study aimed to elucidate the adaptive capacity and resilience of the silkworm gut microbiota in response to different dietary regimens, with a particular focus on identifying key microbial taxa that may contribute to improved host performance and robustness under varying nutritional conditions.

The comparative analysis of alpha and beta diversity indices revealed striking differences in the gut microbiota structure among silkworms reared on different dietary regimens, providing direct evidence for the adaptability and resilience of the silkworm gut microbiota in response to dietary modulations. The SY group exhibited the lowest species richness and diversity, suggesting that the mulberry leaf diet supports a specialized and streamlined gut microbiota adapted to the specific nutritional profile of the leaves. This finding is consistent with previous common knowledge that has reported a distinct and conserved gut microbiota in silkworms fed on mulberry leaves [22,30,31]. The low diversity in the SY group may be attributed to the presence of antimicrobial compounds, such as phenolic glycosides and alkaloids, in mulberry leaves that could selectively inhibit the growth of certain microbial taxa.

Conversely, the SL group, which was administered an artificial diet, housed the most diverse and intricate gut microbiota. This suggests that the artificial diet offers a more varied substrate that promotes a broader spectrum of microbial growth and colonization. The enhanced species richness observed in the SL group could be attributed to the multitude of components integrated into the artificial diet, including soybean meal, corn powder, and an assortment of nutritional additives. These components collectively foster a more diverse microbial community [5,6,12,16]. However, it is crucial to recognize that a higher microbial diversity does not automatically equate to superior host performance. Some microbial taxa, which were found to be more prevalent in the SL group, are known to be opportunistic pathogens or competitive species that could potentially detract from the health and silk productivity of the silkworms [17]. These pathogens have hampered their digestion, and as a result, the silkworms had to adopt an artificial diet instead of natural mulberry.

The ZS group, undergoing a sequential transition from an artificial diet to mulberry leaves, demonstrated a gut microbiota diversity that was intermediate in comparison to the other groups. This result implies that the silkworm gut microbiota has an extraordinary ability to adapt and reorganize in response to alterations in diet. The observed gradual transition in the microbial community’s composition, marked by the co-dominance of the Proteobacteria and Actinobacteria phyla, underscores the resilience and adaptability of the gut microbiota. This capacity for adaptation is vital for silkworms to manage the challenges associated with shifting from an artificial to a natural diet, which encompasses adjustments in nutrient composition, digestibility, and the introduction of plant secondary metabolites [22,32].

The LefSe analysis pinpointed several key microbial taxa that were significantly enriched across the dietary groups, offering valuable insights into their potential functional roles within the silkworm gut. Notably, the ZS group showed enrichment of Proteobacteria, particularly the family Methylobacteriaceae, suggesting their importance in the digestion and metabolism of plant-derived compounds as the silkworms transitioned diets. Methylobacteriaceae are recognized for their role in degrading aromatic compounds and in the biosynthesis of plant growth-promoting substances, including indole-3-acetic acid (IAA) [33]. The significant presence of Methylobacteriaceae in the ZS group indicates their potential role in facilitating the silkworm’s adaptation to a mulberry leaf diet. This could be achieved by enhancing the breakdown of plant cell wall components and aiding in the detoxification of plant secondary metabolites.

The SY group was distinguished by the enrichment of Cyanobacteria and chloroplast-derived taxa, including those from the *Streptophyta* lineage, which are presumably linked to the consumption of mulberry leaves. Consistent with prior research, these taxa are likely to play a role in the digestion of cellulose and other complex plant cell wall polysaccharides. Additionally, they may contribute to the provision of vital nutrients to the silkworm host, such as amino acids and vitamins [7,16,34]. We have confirmed the presence of these taxa with the great importance of the silkworm-mulberry leaf symbiosis in maintaining a healthy and functional gut microbiota.

The SL group presented a unique gut microbiota profile characterized by an increased representation of the Firmicutes and Bacteroidetes phyla, which included the genera *Weissella* and *Lactobacillus*. These particular taxa are renowned for their probiotic attributes and their capacity to ferment an extensive array of carbohydrates. This fermentation process results in the production of short-chain fatty acids (SCFAs), which serve as an energy source for the host [21,35]. The elevated abundance of these taxa within the SL group implies that they may be instrumental in the digestion and metabolic breakdown of the complex carbohydrates found in artificial diets.

Functional profiling of the gut microbiota via the KEGG pathways revealed potential differences in digestive functions across the dietary groups. Notably, the SL group showed an upregulation in ethylbenzene degradation pathways, in contrast to the downregulation of photosynthesis and insulin signaling pathways observed in the SY group. These observations suggest an adaptive shift in the metabolic activities of the gut microbiota to the artificial diet. The increased xenobiotic degradation capability in the SL group may be linked to the presence of plant-derived compounds, such as flavonoids and tannins, which are components of the soybean meal in the artificial diet [7,35,36]. The downregulation of photosynthesis-related pathways in the SL group is consistent with the lower abundance of Cyanobacteria and chloroplast-derived taxa in this group compared to the SY group [6,30,35].

The functional differentiation between the SL and ZS groups, as revealed by the comparative analysis, presented a complex pattern. Notably, there was a downregulation of several KEGG pathways in the ZS group, specifically those associated with meiosis, steroid biosynthesis, and photosynthesis. These adaptive changes likely mirror the gut microbiota’s response to the incremental shift from an artificial to a natural diet, necessitating a reconfiguration of metabolic capabilities to enhance nutrient assimilation and energy generation. Furthermore, the upregulation of pathways related to RNA transport and secondary bile acid biosynthesis in the ZS group implies an augmented capacity for protein synthesis and lipid metabolism. These metabolic enhancements are likely crucial for supporting the silkworm’s growth and development throughout the dietary transition [37].

Despite the study’s contributions, two limitations are noted. First, the experimental design lacks a cohort transitioned from mulberry leaves to an artificial diet, which could provide insights into reverse dietary adaptation. Second, sampling was not performed across all developmental stages, potentially missing critical microbiota shifts. Our future research will aim to address these limitations, enhancing the depth and breadth of our knowledge on the subject.

## 5. Conclusions

Our presented study provides a comprehensive analysis of the gut microbiota structure and functional potential in silkworms subjected to different dietary regimens. The results highlight the remarkable adaptability and resilience of the silkworm gut microbiota in response to dietary modulations, as evidenced by the distinct microbial profiles associated with each dietary group. The identification of key microbial taxa, such as the Methylobacteriaceae, or *Weissella* and *Lactobacillus*, that are differentially enriched across the dietary groups paves the way for the development of targeted microbial interventions to enhance silkworm health and productivity. The development of tailored probiotic supplements or microbial inoculants based on the key taxa identified in this study could potentially improve the silkworm’s resilience to dietary stress, enhance nutrient utilization, and promote overall host health.

## Data Availability

The raw sequencing data in the *.fq format were uploaded and released in the Zenodo database with the doi:10.5281/zenodo.10206330.

## Supplementary Materials

The following supporting information can be downloaded at: https://www.mdpi.com/article/10.3390/ani14091261/s1. Supplement File S1: The statistics for alpha diversity indices. Supplement File S2: The Bray-Curtis distance matrix describes the β-diversity. Supplement File S3: The summarized taxonomical and abundance statistics of all sequencing samples. Supplement File S4: The summarized taxonomical statistics and *p* values produced by LefSe analysis. Supplement File S5: The feature importance results from the Random Forest analysis. Supplement File S6: The enriched KEGG pathways associated with the metabolites show significant differences across the experimental groups. Supplement Figure S1: Rarefaction Curves Across All 30 Sequencing Samples. The horizontal axis represents the sequencing depth, while the vertical axis indicates the number of observed OTUs.
