# Supplementary figures and images for: Gut Microbiota Analysis in Silkworms (Bombyx mori) Provides Insights into Identifying Key Bacterials for Inclusion in Artificial Diet Formulations
Source: Animals (Basel). 2024 Apr 23;14(9):1261. doi: 10.3390/ani14091261 (PMC11083763; doi:10.3390/ani14091261)

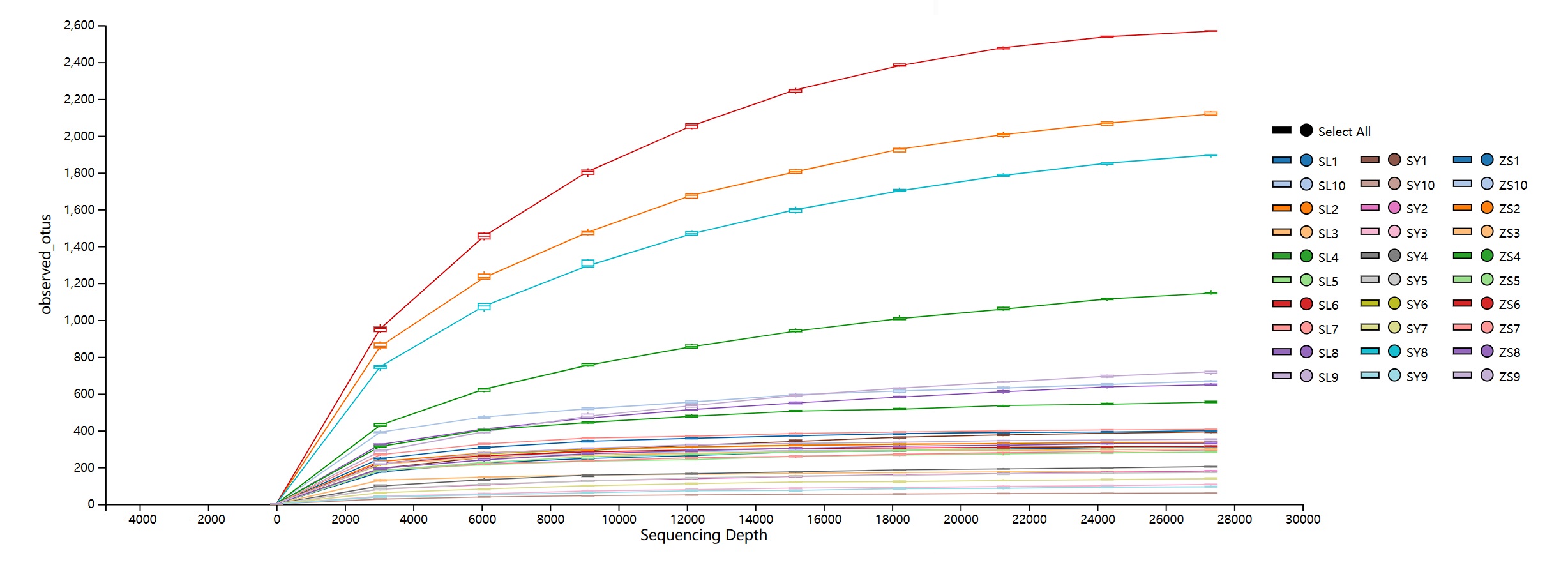

Supplement: Supplementary file 1 [file animals-14-01261-s001.zip › Supplement Figure S1.jpg]
